# Association of Callous Traits with Reduced Neural Response to Others’ Pain in Children with Conduct Problems

**DOI:** 10.1016/j.cub.2013.04.018

**Published:** 2013-05-20

**Authors:** Patricia L. Lockwood, Catherine L. Sebastian, Eamon J. McCrory, Zoe H. Hyde, Xiaosi Gu, Stéphane A. De Brito, Essi Viding

**Affiliations:** 1 Division of Psychology and Language Sciences, University College London, 26 Bedford Way, London WC1H 0AP, UK; 2 Department of Psychology, Royal Holloway, University of London, Egham Hill, Egham, Surrey TW20 0EX, UK; 3 Wellcome Trust Centre for Neuroimaging, University College London, Queen Square, London WC1N 3BG, UK; 4 School of Psychology, University of Birmingham, Edgbaston, Birmingham B15 2TT, UK

## Abstract

Children with conduct problems (CP) persistently violate others’ rights and represent a considerable societal cost [1]. These children also display atypical empathic responses to others’ distress [2], which may partly account for their violent and antisocial behavior. Callous traits index lack of empathy in these children and confer risk for adult psychopathy [3]. Investigating neural responses to others’ pain is an ecologically valid method to probe empathic processing [4], but studies in children with CP have been inconclusive [5, 6]. Using functional magnetic resonance imaging (fMRI), we measured neural responses to pictures of others in pain (versus no pain) in a large sample of children with CP and matched controls. Relative to controls, children with CP showed reduced blood oxygen level-dependent responses to others’ pain in bilateral anterior insula (AI), anterior cingulate cortex (ACC), and inferior frontal gyrus, regions associated with empathy for pain in previous studies [7, 8]. In the CP group, callous traits were negatively associated with responses to others’ pain in AI and ACC. We conclude that children with CP have atypical neural responses to others’ pain. The negative association between callous traits and AI/ACC response could reflect an early neurobiological marker indexing risk for empathic deficits seen in adult psychopathy.

## Highlights

• Children with conduct problems showed reduced neural responses to others’ pain
• Reductions in anterior insula, anterior cingulate cortex, and inferior frontal gyrus
• Callous traits associated with the magnitude of reduction
• Findings may reflect early neurobiological risk for adult psychopathy

## Results

Conduct problems (CP) in children include aggression, theft, and cruelty to others [9]. Children with CP are considerably more likely to engage in antisocial behavior in adulthood than typically developing children and are at risk for developing adult psychopathy [3]. Antisocial behaviors displayed by children with CP may reflect atypical empathic responses to others’ suffering [2]. Empathy is the capacity to understand and resonate with the affective experience of another [10] and plays a key role in inhibiting aggression and promoting prosocial behavior [11, 12]. Callous and unemotional (CU) traits index low empathy in children with CP, as well as diminished guilt, callous use of others, and shallow emotions [13, 14].

One method for investigating neural processing of empathy is to measure responses to others’ pain [4]. Delineating these responses in children with CP is of particular interest because this group often inflicts pain on others [1]. fMRI studies in healthy populations have identified a network of brain regions activated by both the experience and observation of pain. This network includes sensory regions such as somatosensory cortex, affective-motivational regions (linked to processing emotional responses to pain), such as anterior insula (AI) and anterior cingulate cortex (ACC), and cognitive-regulatory regions, such as inferior frontal gyrus (IFG) [7, 8, 10, 15, 16].

Atypical function and structure in several of these regions, including AI, ACC, and prefrontal cortex, have been implicated in the pathophysiology of childhood CP and adult psychopathy [17, 18]. However, to date, only two studies have investigated neural processing of empathic pain in children with CP [5, 6], with inconclusive results. Decety et al. [6] found that, compared with controls, children with CP showed increased neural responses to others in pain in regions including the insula, anterior midcingulate, striatum, and amygdala. Aggressive CP symptoms were positively correlated with IFG, cingulate cortex, amygdala, and periaqueductal gray responses. However, CU traits were not measured, and the CP sample was small (n = 8), making replication and extension of this work important. Another study measured event-related potentials and found reduced responses to others’ pain in children with CP and high levels of CU traits [5]. These findings provide preliminary evidence that children with CP show atypical responses to others’ pain, which may be partially driven by CU traits.

To test the hypothesis that children with CP would show atypical neural responses to others’ pain, we recorded fMRI responses to pictures of others’ hands and feet either in pain or in no pain (from [19]) in a large sample of children with CP (n = 37) and controls (n = 18). Groups were matched for IQ, age, socioeconomic status, and ethnicity. Participants performed an incidental hand/foot judgment task to ensure they were attending to the stimuli. We also acquired parent and teacher ratings of CU traits using the Inventory of Callous-Unemotional Traits (ICU) [20], a standard research measure comprising callous, uncaring, and unemotional subscales. On the basis of previous research suggesting reduced empathy in children with CP [2, 13, 14], we predicted reduced neural responses in three regions of interest (ROI): AI, ACC, and IFG, all linked to empathy for pain in previous studies [7, 8, 10]. We further predicted that callous traits would be negatively associated with AI and ACC response, because response in these regions has been related to affective aspects of empathy and callous traits in particular index poor empathy.

Results from whole-brain analyses for the main effect of Pain > No Pain (and the reverse) and the group by condition interaction are displayed in Table S1 available online (see also Figure S1 and Supplemental Discussion). Main effects were found in regions previously associated with empathy for pain and largely replicated a previous study using the same stimuli [19]. ROI analyses for Pain > No Pain revealed the predicted pattern of reduced response in the CP relative to control group in bilateral AI (*t*[53] = 2.08, p = 0.02), ACC (*t*[53] = 1.66, p = 0.05), and IFG (*t*[53] = 2.45, p < 0.01) (see Experimental Procedures and Supplemental Experimental Procedures for full details of analyses, including ROI definition and statistical thresholds). Levene’s test indicated that variance did not differ between groups for any ROI (p values > 0.20).

We then examined our second hypothesis, that callous traits would be associated with reduced ROI responses to Pain > No Pain within the CP group. On the basis of previous findings showing that CP symptoms and CU traits exert suppressor effects on one another (see [21, 22]), we conducted multiple regressions to investigate unique contributions of ICU subscales (callous, uncaring, unemotional) and CP symptoms to neural responses in our ROIs (see Table S2 for bivariate correlations). One participant was excluded from these analyses due to missing data.

**Figure 1:**
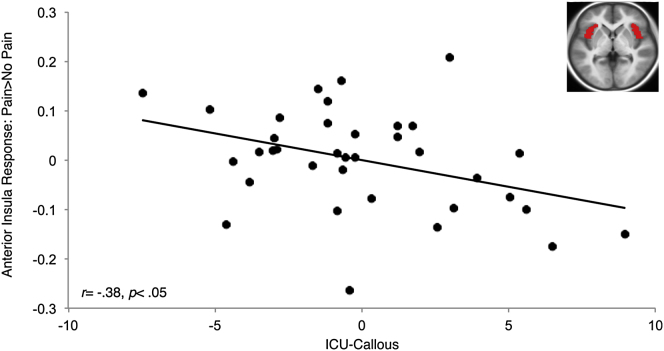
Partial Regression Plot for the CP Group Showing a Negative Relationship between Bilateral AI Response to Pain > No Pain and Unique Variance Associated with ICU-Callous Traits Partial regression plot for the CP group (n = 36) shows a negative relationship between bilateral AI response to Pain > No Pain and unique variance associated with ICU-callous traits after controlling for CASI-CD, ICU-unemotional, and ICU-uncaring scores. Inset shows horizontal section (z = 0) of bilateral AI ROI overlaid on an average T1 structural image from all participants. Bilateral AI response was calculated by averaging left and right AI response. *P* and r reflect partial correlation coefficients.

**Figure 2:**
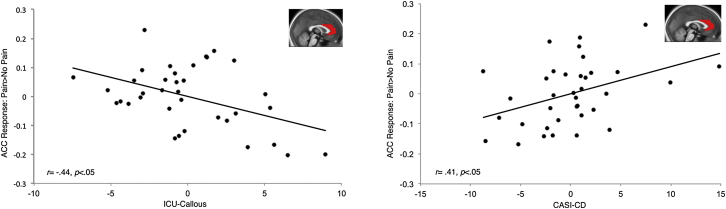
Partial Regression Plots Showing Associations with ACC Response to Pain > No Pain in the CP Group Partial regression plots showing associations with ACC response to Pain > No Pain in the CP group (n = 36). Left: negative relationship between ACC response and unique variance associated with ICU-callous traits, after controlling for CASI-CD, ICU-unemotional, and ICU-uncaring scores. Right: positive relationship between ACC response and unique variance associated with CASI-CD scores, after controlling for ICU-callous, unemotional, and uncaring subscale scores. Insets: sagittal section (x = 0) of ACC ROI overlaid on an average T1 structural image from all participants. Bilateral ACC response was calculated by averaging left and right ACC response. *P* and r reflect partial correlation coefficients.

In AI, a significant negative relationship was found between unique variance associated with callous traits and neural response (β = −0.625, p = 0.029) (Figure 1). Neither CP symptoms nor uncaring or unemotional subscales were associated with AI response (all p values > 0.10). In ACC, a significant negative relationship was found between unique variance associated with callous traits and neural response (β = −0.729, p = 0.010), whereas a positive relationship was found between unique variance associated with CP symptom scores and neural response (β = 0.485, p = 0.019) (Figure 2). No relationships were found in relation to the uncaring or unemotional subscale scores (p values *>* 0.24). In IFG, no associations with unique variance were found. To investigate potential effects of commonly comorbid attention-deficit hyperactivity, generalized anxiety, and depression symptoms, we included these variables in follow-up regression analyses. All significant results remained at p < 0.05, and none of these variables predicted AI or ACC response (all p values > 0.25). In addition, when age was included in follow-up regression analyses, all results remained significant at p < 0.05.

Behavioral data from the incidental hand/foot judgment task showed a main effect of condition for both reaction times (*F*[1,53] = 71.85, p *<* 0.001) and errors (*F*[1,53] = 6.40, p = 0.014), with significantly slower RTs and greater error rates in the pain condition (mean RT = 910 ms, SD = 140 ms; mean % error = 6.82, SD = 5.05) compared with no pain (mean RT = 863 ms, SD = 130 ms; mean % error = 5.63, SD = 4.55). We therefore reran all regression models, controlling for RT and error-rate difference scores (pain − no pain) to exclude the possibility that differing cognitive conflict demands could account for our findings. All results remained significant at p < 0.05. RT and error data showed no main effects of group or interaction between group and condition (see section “Behavioral Data” in Supplemental Results).

## Discussion

We demonstrate reduced neural responses to others’ pain in children with conduct problems compared with matched controls in three regions (bilateral AI, ACC, and IFG) associated with affective-motivational and cognitive-regulatory empathic processing. This is the first fMRI study to investigate empathy for pain processing in a large sample of children with CP, using a well-controlled task matched for visual and social content. We also show a negative association between callous traits and responses in AI and ACC, regions related to unpleasant emotions generated in response to others’ pain [7, 8].

Meta-analyses indicate that AI and ACC are consistently activated during empathy for pain and have a close functional relationship [7, 8, 16]. AI plays an important role in sensory integration [23] and interoceptive awareness [24] and may be involved in awareness of unpleasant feelings during empathy for pain [16]. Interestingly, abnormal AI function and structure have frequently been reported in both children with CP and in adults with psychopathy [6, 21, 25, 26]. However, our observation of reduced AI response is at odds with one study [6], which found increased AI response in children with CP. This could be because in that study [6], pain caused by accident was contrasted with pain caused by others, whereas our pain and no-pain conditions were matched for agency. Increased AI reactivity [6] may reflect differences in agency processing rather than pain processing per se. Differences in the samples between the two studies (e.g., levels of callous traits) may also have contributed to the divergent findings. Our data provide additional support for the view that atypical AI function represents a neural marker of disrupted empathic processing in CP and that AI hypoactivity relates to differences in processing others’ pain.

It has been suggested that ACC mediates responses to unpleasant negative emotion generated in AI [16]. However, the role of ACC in empathy may be more domain general than that of AI, given its involvement in general information processing [19, 27]. Like AI, atypical ACC function in CP has been reported previously, again with mixed findings [6, 28]. One study reported reduced ACC response to negative pictures in CP [28], whereas another found greater ACC response in children with CP to videos of others in pain versus no pain [6]. Our finding provides converging evidence that ACC function is atypical in CP and in particular that there is hypoactivity of response during empathy for pain.

The pattern of reduced IFG response is of interest, given the known involvement of this region in emotion regulation and pain suppression [15, 29, 30] as well as in empathy tasks [7, 8]. It is possible that fewer regulatory resources were required, given that responses in other regions processing empathy for pain were hypoactive. It could also be that the result reflects known deficiencies in emotion regulation in children with CP [31].

To address our second aim, we explored dimensional contributions of CU traits and CP symptoms to ROI responses. As predicted, unique variance associated with callous traits was negatively related to AI and ACC response. Because the callous subscale of the ICU contains items reflecting poor empathy in everyday life, our findings provide evidence of convergent validity between questionnaire and neural measures of empathy in CP. Moreover, callousness is an important feature of adult psychopathy [32], and childhood CU traits predict adult psychopathy [3]. Blunted neural responses to pain in children with higher levels of callous traits may characterize a developmental vulnerability to serious antisocial behavior; for a minority, such a pattern may interact with other vulnerability factors to increase risk of adult psychopathy.

Interestingly, CP symptoms were positively correlated with ACC response. These results complement recent findings [21] showing opposing unique contributions of CU traits and CP symptoms to neural response in the amygdala. Heterogeneity in CP may help to explain inconsistencies across previous studies reporting both increased and decreased ACC responses in CP [6, 28]. Importantly, differences in cognitive conflict (as indexed by RT and error differences between pain and no-pain conditions) did not account for the ACC findings. More generally, these data highlight that children with CP are a heterogeneous group with varying neurocognitive vulnerabilities, with callous traits of particular importance in predicting empathic dysfunction.

Limitations of the current study include the use of a research diagnosis of CP and a focus on males. Replication in a clinically diagnosed sample will be important, as will investigation of potential gender differences. Additionally, our task did not allow us to explore the function of component processes within the empathy for pain response in CP. Future studies should address whether there is a specific aspect of this response that is atypical in CP, e.g., basic arousal, interoceptive processing, or higher-level emotional responses to others’ suffering. Finally, replication and extension of the current study is required. In particular, longitudinal studies documenting the development and persistence of reduced neural responses to others’ pain in children with CP would be informative.

Despite these limitations, our data extend understanding of the neural basis of CP and empathy in several important ways. To our knowledge, this is the first study to investigate empathic pain processing in a large sample of children with CP compared with controls on a task matched for visual and social content. First, we show reduced neural responses to others’ pain in children with CP. Second, we show that callous traits in particular may underlie atypical neural responses to others’ pain in CP, which may represent an early neurobiological marker for later psychopathy. Third, the finding that callous traits and CP symptoms show opposing relationships with ACC response suggests a potential explanation for mixed reports of hyperactivation [6] and hypoactivation [28] of ACC to negative affective stimuli in CP. Clinically, our data may have consequences for empathy training implementation (e.g., in relation to victim empathy [33]) in children with high levels of callous traits. Systematic evaluation of training outcomes should take callous traits into account. It remains an empirical question whether empathic responding can be normalized in children with CP (and varying levels of callous traits) or whether behavioral equivalence is better achieved through compensatory strategies that leverage spared cognitive processes [13, 14].

## Experimental Procedures

### Participants

Right-handed boys aged 10–16 (mean [SD]: controls = 13.68 [1.68]; CPs = 14.05 [1.69]) were recruited from the community via advertisements and local schools. Screening questionnaires were completed by 143 parents and teachers. CP was assessed using the Child and Adolescent Symptom Inventory (CASI-4R) [34] Conduct Disorder scale (CASI-CD). CASI-CD cutoff scores for inclusion in the CP group were: parent report = 4+ (ages 10–12) and 3+ (ages 12–16) or teacher report = 3+ (ages 10–12), 4+ (ages 12–14), and 6+ (ages 15–16). These scores are associated with a clinical diagnosis of CD [35]. CU traits were assessed using the Inventory of Callous-Unemotional Traits (ICU) [20]. Total scores for the three ICU subscales (callous, uncaring, and unemotional) were calculated [20]. Both CASI-CD and ICU were scored by taking the highest ratings from either the parent or teacher questionnaire for each item [36]. For two children with CP, only parent ratings were available. The Strengths and Difficulties Questionnaire (SDQ [37]) was used to screen for psychopathology (hyperactivity, CP, emotional symptoms, peer problems) in the controls. All control participants scored below the CP group median on the ICU and in the normal range on the CASI-CD and SDQ. For both groups, exclusion criteria included previous diagnosis of neurological or psychotic disorder, including autism spectrum disorders, and current prescription for psychiatric medication (all children were unmedicated). Written informed consent from parents and written assent from participants was obtained.

Fifty-eight children were scanned (39 CPs, 19 controls), with usable data from 37 CPs and 18 controls. Exclusions were due to scanner refusal (1 CP) and teacher questionnaire data obtained after scanning indicating that the child no longer met group criteria (1 CP, 1 control). Groups were matched on IQ, age, ethnicity, and socioeconomic status (Table 1).

**Table 1:** Participant Characteristics

Characteristics and Questionnaires	Group		p Value^a^
	Controls (n = 18)	CP (n = 37)	
**Demographic Variables**			

Age^b^	13.68 (1.68)	14.05 (1.69)	0.456
Socioeconomic Status^b^	3.07 (1.01)	3.23 (1.26)	0.635
F-IQ^c^^,^^d^	102.83 (11.69)	101.17 (13.46)	0.656
V-IQ^c^^,^^d^	53.06 (8.73)	49.92 (10.96)	0.295
P-IQ^c^^,^^d^	49.67 (8.61)	50.33 (9.57)	0.804
Ethnicity^b^^,^^e^	13:2:2:1	26:3:6:2	1.00

**ICU**^f^	24.17 (4.85)	42.97 (10.67)	<0.001

**Child and Adolescent Symptom Inventory**			

Conduct Disorder^f^	0.56 (0.70)	10.14 (6.18)	<0.001
ADHD^d^^,^^g^	9.47 (7.47)	25.04 (13.75)	<0.001
Generalized Anxiety Disorder^d^^,^^g^	2.71 (3.07)	7.46 (5.37)	0.001
Major Depressive Episode^d^^,^^g^	2.61 (1.09)	6.38 (5.40)	0.005

**AUDIT**^c^^,^^d^	1.22 (1.99)	2.11 (3.88)	0.366
**DUDIT**^c^^,^^d^^,^^h^	0.17 (0.51)	2.50 (6.62)	0.143

Abbreviations: CP, conduct problems; F-IQ, full IQ score from the two-subtest Wechsler Abbreviated Scale of Intelligence; V-IQ, verbal IQ score; P-IQ, matrix reasoning IQ score; ICU, Inventory of Callous-Unemotional Traits; ADHD, attention-deficit/hyperactivity disorder; AUDIT/DUDIT, Alcohol/Drug Use Disorders Identification Test.

a All p values were obtained using t tests except for ethnicity (Fisher's exact test used).

b Measures taken at screening phase—parent report.

c Child at scanning session.

d Missing data from 1 CP.

e Ethnicity: White:Black:Mixed:Asian.

f Meaures taken at screening phase—parent and teacher report.

g Measures taken at scanning session—parent report.

h The Drug Use Disorders Identification Test requires participants to rate the frequency of any substance use on a five-point scale from “never” to “almost daily.” The list of drugs includes cannabis, amphetamines, cocaine, opiates, hallucinogens, solvents, and GHB, as well as medicines used in an abusive way.

### Experimental Task

Stimuli were 192 digital photographs showing another person’s hand or foot in painful or nonpainful situations [19]. “Pain” and “No Pain” stimuli (96 pictures per condition) were matched on physical properties and were validated as eliciting empathy-related activations in a previous study [19]. Stimuli were presented in pain and no-pain blocks lasting 20 s and consisting of eight images, each displayed for 2,000 ms with a 500 ms interstimulus interval. Blocks were pseudorandomized, with the same block type never presented more than twice in a row. A fixation cross was presented for 15 s every six blocks.

To ensure attention, participants performed a hand/foot key press judgment on every trial. Participants practiced outside the scanner with painful and nonpainful images not seen in the main experiment, until ≥80% accuracy was reached.

### Psychometric and Questionnaire Measures

Participants completed the Wechsler Abbreviated Scale of Intelligence two-subtest version [38] and the Alcohol and Drug Use Disorders Identification Tests [39, 40]. A parent or guardian completed the CASI-4R scales for symptoms commonly comorbid with CP, including ADHD, generalized anxiety disorder, and major depressive episode (Table 1).

### fMRI Data Acquisition and Analysis

A Siemens Avanto 1.5 T MRI scanner was used to acquire 189 multislice T2^*^-weighted echo planar volumes with blood oxygenation level-dependent contrast (one run of 9 min). The sequence was based on Weiskopf et al. [41] (see Supplemental Information for acquisition parameters, preprocessing pipeline, and procedures for removing data corrupted by participant motion). A 5.5 min T1-weighted MPRAGE scan was acquired for coregistration, normalization, and overlay, and fieldmaps were acquired for unwarping. Data were analyzed using Statistical Parametric Mapping (SPM8; http://www.fil.ion.ucl.ac.uk/spm).

After standard preprocessing, a block analysis compared neural activity associated with pain and no-pain conditions. Regressors included Pain and No Pain (blocks of 20 s duration) and fixation (15 s), modeled as boxcar functions convolved with a canonical hemodynamic response function. The six realignment parameters were also modeled as effects of no interest.

At the first level, Pain > No Pain and No Pain > Pain contrasts were created. Contrast images were entered into second-level analyses, where group (CP versus control) served as a between-subjects variable in independent sample t tests. Main effects are reported at p < 0.05, family-wise error (FWE) corrected across the whole brain, whereas regions from a whole-brain analysis showing a condition by group interaction are presented at p < 0.005, k ≥ 10, uncorrected (no interaction results survived FWE correction across the whole brain) (Table S1). We investigated the condition by group interaction in three a priori regions of interest (bilateral AI, ACC, and IFG). ROIs were anatomically defined using masks from the automated anatomical labeling atlas [42]. The MarsBaR toolbox (http://marsbar.sourceforge.net) was used to calculate mean contrast estimates across bilateral ROIs. Group differences were assessed at a standard statistical threshold of p < 0.05 [43, 44].

## Supplemental Information

Document S1. Figure S1, Tables S1 and S2, Supplemental Results, Supplemental Discussion, and Supplemental Experimental Procedures
