# Supplementary material for: Association of Callous Traits with Reduced Neural Response to Others’ Pain in Children with Conduct Problems
Source: Curr Biol. 2013 May 20;23(10):901–5. doi: 10.1016/j.cub.2013.04.018 (PMC3918856; doi:10.1016/j.cub.2013.04.018)
Supplement: Document S1. Figure S1, Tables S1 and S2, Supplemental Results, Supplemental Discussion, and Supplemental Experimental Procedures [file mmc1.pdf]

Current Biology, Volume *23*
Supplemental Information

Association of Callous Traits with Reduced Neural Response to Others' Pain in Children with Conduct Problems

Patricia L. Lockwood, Catherine L. Sebastian, Eamon J. McCrory, Zoe H. Hyde, Xiaosi Gu, Stéphane A. De Brito, and Essi Viding

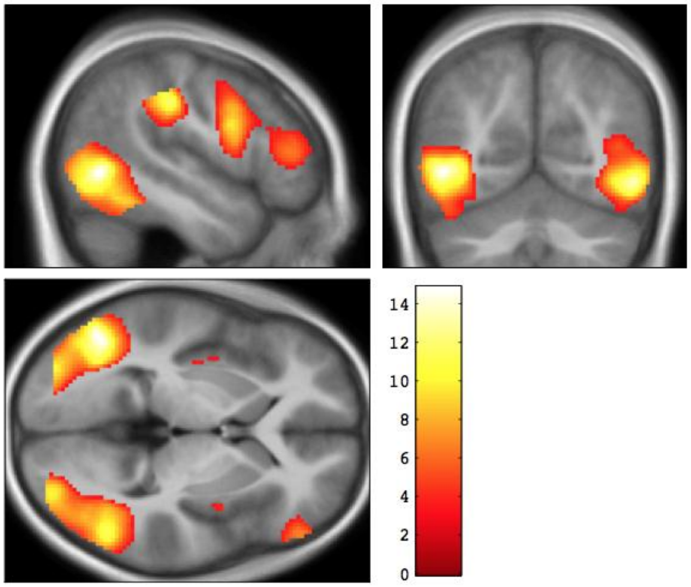

Figure S1. Whole Brain Figure Displayed at Coordinate -48 -62 0 for Contrast Pain>No Pain Overlaid on an Average T1 Structural Image from All Participants

Table S1. Whole Brain Analyses Showing Main Effects across All Participants for Pain>No Pain and the Reverse and Group x Condition Interaction Effects

Brain Region	BA	L/R	Peak voxel			k	t	z	FWE-corrected p-value
Pain>No pain									
Middle temporal gyrus	37	L	-48	-62	0	3817	14.83	>8.00	<.001
ext. middle occipital gyrus	19	L	-34	-86	-2		9.99	7.46	
ext. fusiform gyrus/inferior temporal gyrus	37	L	-46	-48	-16		9.41	7.18	
Secondary somatosensory cortex	40	L	-60	-30	34	1817	13.46	>8.00	<.001
ext. primary somatosensory cortex	2	L	-52	-26	36		12.44	>8.00	
Secondary somatosensory cortex	40	L	-60	-30	24		11.22	>8.00	
Inferior temporal gyrus	19	R	46	-56	-8	3739	13.22	>8.00	
ext. middle temporal gyrus	39	R	52	-62	-6		13.2	>8.00	<.001
ext. middle occipital gyrus	19	R	36	-80	8		10.93	>8.00	
IFG/DLPFC	9	L	-52	6	34	1433	10.09	7.51	<.001
ext. insula (middle)	13	L	-36	-4	14		5.48	4.86	
Primary somatosensory cortex	2	R	62	-22	30	1385	9.29	7.12	<.001
ext. secondary somatosensory cortex	40	R	36	-34	38		5.29	4.72	
DLPFC	46	R	52	40	4	2137	7.95	6.42	<.001
ext. frontal operculum	44/9	R	52	8	26		7.22	6.00	
IFG (triangularis)	45	L	-50	38	10	595	6.31	5.43	.002
Supplementary motor area	6	L	-30	-6	48	167	6.12	5.30	.003
Uncus/fusiform gyrus	20	L	-32	-2	-38	43	5.36	4.77	.03
No Pain>Pain									
Middle temporal gyrus	20	L	-58	-34	-10	414	5.70	5.01	.01
Caudate nucleus	-	R	14	24	4	795	5.69	5.00	.01
Cerebellum	-	L	-42	-66	-38	135	5.60	4.94	.01
Lingual gyrus	18	R	12	-74	-4	802	5.37	4.78	.03
Group x Condition Interaction									p-value (peak uncorrected)
Control>CP (for Pain>No Pain)									
STG/insula (posterior)	22/13	L	-44	-16	-6	91	4.36	4.01	<.001
Cerebellum	-	R	0	-48	-20	74	3.82	3.57	<.001
Middle temporal gyrus	21	R	44	6	-36	24	3.42	3.23	.001
Caudate	-	R	10	10	10	22	3.34	3.17	.001
Supplementary motor area	6	L	-10	12	52	29	3.32	3.15	.001
IFG (orbitalis)	47	L	-22	10	-20	116	3.32	3.15	.001
ext. insula (anterior)	13	L	-32	12	-16		3.05	2.91	.002
Middle frontal gyrus *ext. IFG*	10	L	-36	34	20	266	3.23	3.07	.001
Thalamus	-	L	-4	-14	20	19	3.22	3.06	.001
Cerebellum	-	R	28	-58	-30	50	3.22	3.06	.001
Insula (posterior)	13	L	-28	-30	14	16	3.22	3.06	.001
Cerebellum	-	R	14	-72	-32	30	3.18	3.03	.001
SFG	10	R	22	48	22	38	3.17	3.02	.001
Globus pallidus	-	L	-16	-8	-4	18	3.15	3.00	.001
Brainstem/substantia nigra	-	R	10	-20	-16	25	3.07	2.93	.002
IFG (triangularis)	47	R	54	38	0	24	3.02	2.89	.002
Insula (anterior)	13	L	-30	16	2	29	3.00	2.87	.002
Anterior cingulate	24	L	0	20	24	14	2.92	2.80	.003
Precuneus	31	L	-4	-62	28	13	2.82	2.71	.003
CP>Control (for Pain>No Pain)									
Parahippocampal gyrus	-	L	-18	-20	-16	15	3.22	3.06	.001
Cerebellum	-	L	-2	-48	-4	17	3.08	2.94	.002

Main effects thresholded at $P<.05$ FWE-corrected at the peak level. Group x Condition interaction thresholded at $P<.005$ uncorrected, $k\geq10$.
BA=Putative Brodmann area; L/R=Left/Right; k=cluster size in $2mm^3$ voxels; DLPFC=Dorsolateral prefrontal cortex; IFG=Inferior frontal gyrus; SFG = Superior frontal gyrus; STG=Superior temporal gyrus; ext.=cluster extends into additional region.

Table S2. Bivariate Correlations between Neural Responses to Pain>No Pain in ROIs and Questionnaire Measures of CP Symptoms and Callous, Unemotional, and Uncaring Traits in the CP Group

	Callous traits	Uncaring traits	Unemotional traits	CP symptoms	ADHD	GAD	MDE	Anterior Insula response	Anterior Cingulate response
Uncaring traits	.732**								
Unemotional traits	.683**	.558**							
CP symptoms	.588**	.495**	.558**						
ADHD	.434**	.390*	.279	.425**					
GAD	.227	.205	.158	.517**	.793**				
MDE	.122	.068	.168	.446**	.540**	.801**			
Anterior Insula response	-.048	.082	.232	.266	-.11	-.003	-.046		
Anterior Cingulate response	-.101	.095	.134	.308	-.139	-.011	.133	.618**	
Inferior Frontal Gyrus response	.273	.258	.436**	.398*	-.112	-.119	-.016	.633**	.565**

CP = Conduct problems; ADHD = Attention deficit hyperactivity disorder; GAD = Generalized anxiety disorder; MDE = Major depressive disorder.
** <0.01
* <0.05

Supplemental Results

Behavioural Data

Mean reaction times (RTs) and percentage error rates were calculated. For mean RTs, a group (CP vs. control) by condition (pain, no pain) ANOVA showed no main effect of group ($F(1,53) = .02$, $p = 0.89$) but a main effect of condition ($F(1,53)=71.85$, $p <.001$) with significantly slower RTs when classifying hands and feet in the pain condition (910.08, SD=140.15) compared to no pain (862.72, SD = 129.73). There was no interaction between group and condition ($F(1,53) <.001$, $p > 0.99$). Error data showed a marginal main effect of group ($F(1,53) = 3.17$, $p = 0.08$) with a trend for more errors in the CP than control group, and a main effect of condition ($F(1,53)=6.40$, $p = 0.014$) with significantly more errors when classifying hands and feet in pain compared to no pain (6.82, SD=5.05 vs. 5.63, SD=4.55). There was no group by condition interaction ($F(1,53)=.061$, $p = 0.81$).

Bivariate Correlations between fMRI Data and Questionnaire Measures

Bivariate correlations were conducted within the CP group between neural responses to pain>no pain in regions of interest (AI, ACC, IFG) and CP symptoms and ICU subscales (callous, unemotional and uncaring traits) (Table S2). In the AI and ACC, no significant bivariate relationships were found (all ps>0.07). In IFG, positive correlations were found between neural response and both CP symptoms ($r = .40$, $p = 0.02$), and unemotional traits ($r = .44$, $p < 0.01$). There was no significant R-squared change when adding CP symptoms after unemotional traits, or vice-versa, into a regression model indicating that common variance between CASI-CD and unemotional traits drove the positive relationship with IFG.

Supplemental Discussion

Other notable regions outside our ROIs were hypoactivated in the CP compared to control group. For example, we observed reduced responses in the supplementary motor area (SMA) and posterior insula at an uncorrected threshold (see table S1). It is interesting that these regions have been linked to pain as experienced by the self [5] such as pain intensity and motor preparation [6], consistent with a view that children with CP were experiencing less pain themselves when viewing the pictures. Such a hypothesis would be in line with Cheng et al. [7] who reported that children with CP and high CU traits had increased pain thresholds. However, the role of SMA and posterior insula regions in empathy for pain is currently debated [3] and such an explanation remains tentative. Future studies are required to investigate further the neural basis of self pain processing in CP (provided this could be done in an ethically feasible manner), which would help shed light on the mechanisms underlying reduced affective responses to the suffering of others.

Supplemental Experimental Procedures

fMRI Acquisition and Analysis

Functional sequence acquisition parameters were as follows: 35 2mm slices acquired in an ascending trajectory with a 1mm gap; TE=50ms; TR=2975ms; slice tilt=−30° (T>C); flip angle=90°; field of view=192 mm; matrix size=64x64.

The preprocessing pipeline was as follows: the first 5 and last 2 volumes were discarded. Data were then realigned, unwarped using a fieldmap, normalized via segmentation of participants' structural scans, written with a voxel size of 2x2x2mm, and smoothed with an 8mm Gaussian filter.

To minimize any effects of motion corruption of our data we followed a number of procedures. Firstly we spent some time preparing participants for scanning with the use of a short slide show of brain scans taken when previous participants had either stayed still or moved varying amounts. Participants were also given a short practice/localizer scan, after which feedback was given on how still they kept. After estimation of the realignment parameters, we ran a script to search for motion of more than 1mm (x,y and z directions) or 1 degree (pitch, roll, yaw) in any direction between acquisition of one volume and the next. Volumes flagged by the script (as well as surrounding volumes) were then inspected visually for motion artifacts. For a few random participants, the whole time series was inspected for motion artifacts to check the validity of the threshold chosen in the script. On the basis of previous studies from our laboratory [1,2] we decided a priori to exclude any participants where more than 10% of the volumes were corrupted by motion artifacts. No participants reached this threshold.

For 14 participants (11 CPs, 3 controls), extra regressors were included within the first-level analysis design matrix to model any images corrupted by motion. These images were removed and the adjacent images interpolated to prevent distortion of the between-subjects mask. Data were high-pass filtered at 128 seconds to remove low-frequency drifts. For one CP, half of the fMRI time series (91 scans) was discarded due to the participant falling asleep midway through scanning.

ROI Selection

We included hemisphere as a separate factor in repeated measures ANOVAs for each ROI. These showed no significant differences between hemispheres for the condition*group interaction effect of interest for any ROI (ps>.40). Since we had no a priori hypothesis regarding laterality, we therefore collapsed data across bilateral ROIs. We selected the anterior portion of the insula by modifying the aal atlas mask to include all voxels y>0 on the basis of several previous studies suggesting that it is the anterior portion of the insula that is involved in affective-motivational aspects of empathic pain processing [3,4]. For ACC we used the standard ACC aal mask. The peak co-ordinate from Lamm et al. [3] falls within this mask in the left hemisphere and falls on the border of the mask in the right hemisphere. The IFG ROI was selected as pars triangularis aal mask as this was the portion of the IFG where the most consistent peak activation was located in the meta-analyses of Fan et al. [4].

Supplemental References

1. Sebastian, C., McCrory, E.J.P., Cecil, C.A.M., Lockwood, P., De Brito, S. A., Fontaine, N.M.G., and Viding, E. (2012). Neural responses to affective and cognitive theory of mind in children with conduct problems and varying levels of callous-unemotional traits. Arch Gen Psychiatry *69*, 814-822.
2. McCrory, E., De Brito, S. A., Sebastian, C., Mechelli, A., Bird, G., Kelly, P., and Viding, E. (2011). Heightened neural reactivity to threat in child victims of family violence. Curr Biol. *21*, R947-R948.
3. Lamm, C., Decety, J., and Singer, T. (2011). Meta-analytic evidence for common and distinct neural networks associated with directly experienced pain and empathy for pain. Neuroimage *54*, 2492-2502.
4. Fan, Y., Duncan, N.W., de Greck, M., and Northoff, G. (2011). Is there a core neural network in empathy? An fMRI based quantitative meta-analysis. Neurosci Biobehav Rev. *35*, 903-911.
5. Singer, T., Seymour, B., O'Doherty, J., Kaube, H., Dolan, R. J., and Frith, C. D. (2004). Empathy for pain involves the affective but not sensory components of pain. Science *303*, 1157–1162.
6. Apkarian AV, Bushnel MC, Treede RD and Zubieta JK. Human brain mechanisms of pain perception and regulation in health and disease. Eur J Pain 2005. *9,* 463–84.
7. Cheng, Y., Hung, A.Y., and Decety, J. (2012). Dissociation between affective sharing and emotion understanding in juvenile psychopaths. Dev Psychopathol. *24*, 623-636.
